# A Systematic Review and Meta-Analysis of the Efficacy of Kinesio Taping for Pain Management and Pressure Pain Threshold in Myofascial Pain Syndrome

**DOI:** 10.1155/prm/8881632

**Published:** 2025-08-18

**Authors:** Mahmoud Kandeel, Mohamed Marzok, Sheryar Afzal, Ahmed Meligy, Maryam Mahmoud, Ibrahim Albokhadaim, Khalid M. Al Khodair, Sameer Alhojaily

**Affiliations:** ^1^Department of Biomedical Sciences, College of Veterinary Medicine, King Faisal University, Al-Hofuf, Al-Ahsa, Saudi Arabia; ^2^Department of Clinical Sciences, College of Veterinary Medicine, King Faisal University, Al-Hofuf, Al-Ahsa, Saudi Arabia; ^3^College of Medicine, Delta University for Science and Technology, Gamasa, Dakahlia, Egypt; ^4^Department of Anatomy, College of Veterinary Medicine, King Faisal University, Al-Hofuf, Al-Ahsa, Saudi Arabia

**Keywords:** kinesio taping, meta-analysis, musculoskeletal disorders, myofascial pain syndrome, pain management, pressure pain threshold, randomized controlled trials

## Abstract

Myofascial pain syndrome (MPS) is a prevalent musculoskeletal disorder characterized by myofascial trigger points (MTrPs), which can significantly impact an individual's quality of life. This study aimed to evaluate the efficacy of Kinesio taping (KT) in reducing pain intensity and increasing pressure pain threshold (PPT) in individuals with MPS. A comprehensive search was performed across five electronic databases (PubMed, Web of Science, Cochrane Library, Embase, and SCOPUS) from inception to May 2024. Randomized controlled trials (RCTs) comparing KT to a control group, including no intervention, placebo, or sham taping, in individuals with MPS were included. Data on pain intensity and PPT were extracted and pooled using RevMan 5.4 software. A total of 15 RCTs were included in the systematic review and meta-analysis. The pooled analysis showed a significant reduction in pain intensity in the KT group compared to the control group immediately after intervention (mean difference [MD] = −1.07, 95% confidence interval (CI) [−1.93, −0.20], *p* = 0.02), within the first week (standardized mean difference [SMD] = −1.44, 95% CI [−2.39, −0.49], *p* = 0.003), and after 2-3 weeks (SMD = −0.97, 95% CI [−1.46, −0.49], *p* < 0.0001). However, the effect diminished after 4–6 weeks (MD = −0.90, 95% CI [−1.65, −0.14], *p* = 0.02). Regarding PPT, KT significantly increased PPT within the first week (MD = 4.32, 95% CI [2.47, 6.16], *p* < 0.00001) but not immediately after intervention or after 2-3 and 4–6 weeks. This meta-analysis provides evidence that KT is effective in reducing pain intensity and increasing PPT in individuals with MPS, particularly in the immediate and short-term periods. However, the effects on pain reduction and PPT diminish over time, suggesting a need for reapplication or combination with other interventions for sustained long-term benefits.

## 1. Introduction

Myofascial pain syndrome (MPS) is a musculoskeletal disorder that is characterized by the existence of myofascial trigger points (MTrPs), which are hyperirritable and painful points situated in skeletal muscle fibers causing pain and discomfort [1]. These points are associated with localized and referred pain, muscle weakness, and limitation of the movements, which could significantly impact an individual's quality of life and functional status [2]. Trigger points develop within skeletal muscles, commonly triggered by factors such as muscle overuse, injury or poor posture resulting in localized pain and tenderness [3]. MPS is a prevalent condition, affecting a substantial portion of patients with pain, estimated to be 30% [4]. About 88.9% of patients with chronic nonspecific neck pain had at least one trigger point, indicating a high prevalence of MPS in this population [5].

The therapy of MPS can involve different kinds of therapy aimed at pain relief, muscle rehabilitation and improvement of the quality of life [6]. Conventional treatments for MPS encompass stretching exercises, heat therapy, electrical stimulation, massage, manual manipulation, trigger point injections, acupuncture, medication, and more recently Kinesio taping (KT) [7]. KT is a form of therapeutic taping that has been developed by a Japanese physiotherapist, Dr. Kenzo Kase, in the early 1970s and is a type of adhesive tape that is stretched and applied over the skin and muscle in the direction of the muscle fibers or over the area that requires taping [8]. The suggested mechanism of KT is by lifting the skin leading to improved blood flow and lymph drainage, increasing the subcutaneous space and activating the muscles, which might help in reducing pain and increasing muscle strength [7, 8].

Pressure pain threshold (PPT) is the amount of pressure the subject can endure before he or she feels pain, which is the point at which pressure sensation turns into pain [9, 10]. PPT assessment may facilitate to identification of pain processing pathways and can be applied for the assessment and management of various MSK disorders including MPS [11].

KT has been proven to be effective in the treatment of MPS, and this has been a subject of discussion in various studies with different degrees of evidence [12]. The potential efficacy of KT in the management of MPS has been the subject of numerous research studies, with varying degrees of evidence supporting their use. While some studies have reported positive outcomes, such as reduced pain intensity, improved range of motion (ROM), and enhanced muscle function, others have yielded conflicting or inconclusive results [13–15].

In the context of MPS, a meta-analysis on the effectiveness of KT and PPT can serve as a valuable resource for healthcare professionals, guiding evidence-based decision-making and informing clinical practice guidelines. By synthesizing the available evidence, this study aims to provide a comprehensive understanding of the role of KT in the management of MPS, specifically regarding pain intensity and PPT.

## 2. Method

The meta-analysis followed the standards outlined in the Cochrane Handbook for Systematic Reviews and Meta-Analyses [16] and adhered to Preferred Reporting Items for Systematic Reviews and Meta-Analyses (PRISMA) guidelines [17]. All meta-analysis steps were conducted independently by two authors, with any disagreements resolved through consensus or consultation with a third senior author.

### 2.1. Search Strategy

A comprehensive search was performed across five electronic databases: PubMed, Web of Science, Cochrane Library, Embase, and SCOPUS from start to May 2024. The identified sources matched the keywords, and Medical Subject Heading (MeSH) terms associated with KT and MPS. The study protocol was recorded at the PROSPERO register (accession number CRD42024551103).

### 2.2. Study Selection Criteria

Studies were included if they met the following criteria: (1) only RCTs; (2) studies published in English; (3) studies with human participants diagnosed with MPS; (4) studies comparing the KT intervention arm with a control group, which may include no intervention, placebo, or sham taping; (5) studies reporting on pain intensity and PPT; and (6) studies providing sufficient data for meta-analysis. Studies were excluded if they involved KT combined with other interventions or if they compared KT with an active treatment. The screening process consisted of two distinct stages: title and abstract screening followed by full-text screening. Additionally, a manual screening of the references of the included studies was conducted.

### 2.3. Data Extraction

Data extracted from the studies included age and gender of the study participants, types of KT interventions, type of control group, outcomes measured, and follow-up time. Additionally, baseline data encompassing body mass index (BMI), pain scores, and PPT were collected. Furthermore, outcomes were extracted for pain and PPT at various endpoints, including assessments immediately postintervention, within the first week, at 2-3 weeks, and at 4–6 weeks following the intervention.

### 2.4. Quality Assessment

The quality of methodological aspects of the included studies was evaluated using the “Revised Cochrane Risk of Bias” tool [18]. All the studies were assessed for bias in five areas of the randomization process, the intervention, the outcome, the measurements of the outcome, and the selection of the results presented.

### 2.5. Data Synthesis and Statistical Analysis

Meta-analysis was conducted Review Manager software (RevMan Version 5. 4). The data were pooled as mean difference (MD)/standardized mean difference (SMD) accompanied by a 95% confidence interval (CI). Heterogeneity between studies was assessed using the Cochran's *Q* test and *I*^2^ statistic. We employed the fixed-effect model for homogeneous data and the random-effects model for heterogeneous data, utilizing the leave-one-out test when the *p*-value was less than 0.1 and *I*^2^ was greater than 50%.

## 3. Results

**Figure 1:**
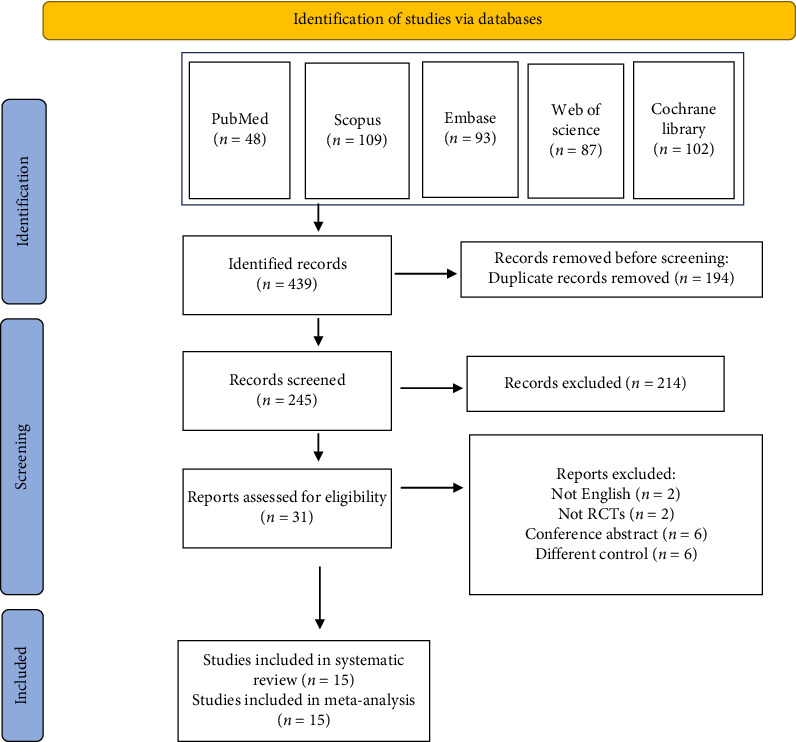
PRISMA flow diagram.

The search for relevant studies identified a total of 439 records from five databases: PubMed (48), SCOPUS (109), Embase (93), Web of Science (87), and Cochrane Library (102). Out of 439 records, 194 records were duplicates and were excluded; 245 records were left for title and abstract screening. Out of these, 214 were excluded and 31 reports were identified for full-text screening. In this stage, 16 reports were excluded for the following reasons: language other than English (2 reports), not randomized controlled trials (RCTs) (2 reports), conference abstracts (6 reports), and different control groups (6 reports). In total, 15 papers were included in the systematic review and meta-analysis [13, 14, 19–31] (Figure 1).

### 3.1. Summary and Baseline of the Included Studies

Most studies used sham taping as a control and focused on the upper trapezius muscle. The shapes varied from star, Y, I, X, and diamond shapes, with applications ranging from single sessions to multiple applications over days or weeks. Pain was commonly assessed using the VAS or the NRS. Across the studies, mean values for age range for KT from 20.6 to 44.80 years, BMI from 20.30 to 28.96, pain scores (VAS/NPS/NRS) from 1.39 to 8.21, and PPT from 0.73 to 33.4. Further details are shown in Tables 1 and 2.

### 3.2. Quality Assessment

**Figure 2:**
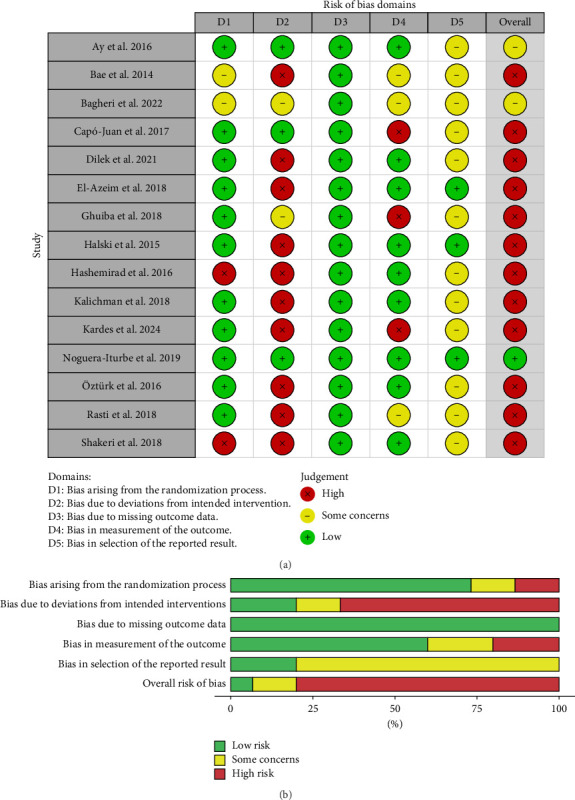
Risk of bias assessment. (a) Risk of bias graph. (b) Risk of bias summary.

Noguera-Iturbe et al. show low bias in all the domains analyzed [28]. Two of the included RCTs were considered to have some concerns because they lacked protocol, details of the outcome assessment, or deviation from the intended interventions were not described in detail [22, 30]. The other studies were considered to have a high risk of bias primarily due to failure to blind both the patients and the professionals (Figures 2(a) and 2(b)).

### 3.3. Outcomes

#### 3.3.1. Change in Pain Score

##### 3.3.1.1. Immediately After Intervention (VAS Score)

**Figure 3:**
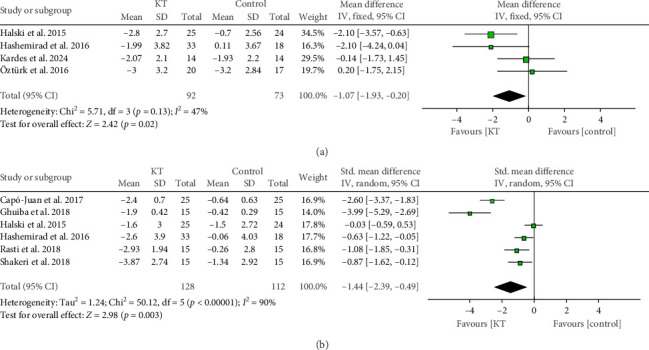
Forest plot of the change in VAS score. (a) Forest plot of the change in VAS score immediately after intervention. (b) Forest plot of the change in pain score in the first week.

The pooled analysis of 4 RCTs showed a significant reduction in the postoperative VAS score in the KT group immediately after the intervention compared with the control group (MD = −1.07, 95% CI [−1.93, −0.20], *p*=0.02), and the data were homogeneous (*p*=0.13, *I*^2^ = 47%) (Figure 3(a)).

##### 3.3.1.2. In the First Week

In the first week, the pooled analysis of 6 RCTs showed a significant reduction in the pain score in the KT group compared with the control group (SMD = −1.44, 95% CI [−2.39, −0.49], *p*=0.003), but the data showed unresolvable heterogeneity (*p* < 0.00001, *I*^2^ = 90%) (Figure 3(b)).

##### 3.3.1.3. After 2-3 Weeks

**Figure 4:**
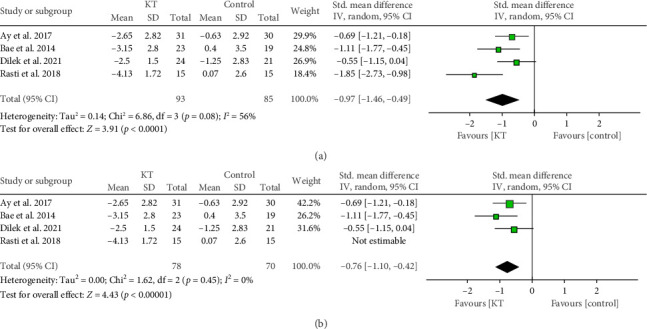
Forest plot of the change in pain score in the second week. (a) Before resolving heterogeneity. (b) After resolving heterogeneity.

Similarly, the pooled analysis of 4 RCTs showed a significant reduction in the pain score in the KT group after 2-3 weeks compared with the control group (SMD = −0.97, 95% CI [−1.46, −0.49], *p* < 0.0001), but the data were heterogeneous (*p*=0.08, *I*^2^ = 56%). This heterogeneity was resolved by excluding Rasti et al. (*p*=0.45, *I*^2^ = 0), and the results remained significant (SMD = −0.76, 95% CI [−1.10, −0.42], *p* < 0.00001) (Figures 4(a) and 4(b)).

##### 3.3.1.4. After 4–6 Weeks (VAS Score)

**Figure 5:**
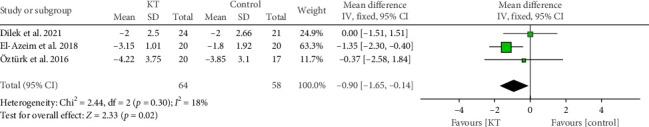
Forest plot of the change in VAS score after 4–6 weeks.

After 4–6 weeks, the pooled analysis of 3 RCTs showed a significant reduction in the pain score in the KT group compared with the control group (MD = −0.90, 95% CI [−1.65, −0.14], *p*=0.02), but the data showed unresolvable heterogeneity (*p*=0.30, *I*^2^ = 18%) (Figure 5).

#### 3.3.2. Change in PPT (kg/cm^2^)

##### 3.3.2.1. Immediately After Intervention

**Figure 6:**
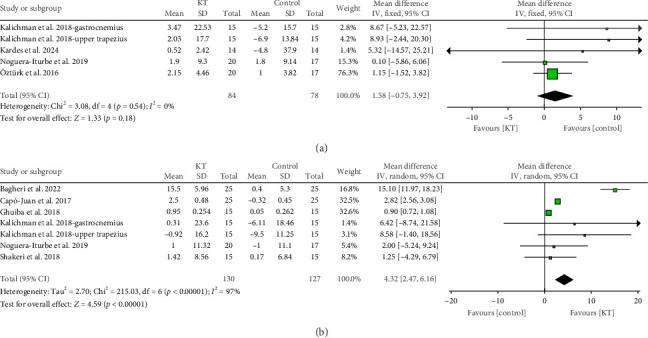
Forest plot of the change in PPT (kg/cm^2^). (a) Forest plot of the change in PPT (kg/cm^2^) immediately after intervention. (b) Forest plot of the change in PPT (kg/cm^2^) in the first week.

There was an insignificant difference between the KT and control groups in the change in PPT immediately after intervention (MD = 1.58, 95% CI [−0.75, 3.92], *p*=0.18), and the results were homogeneous (*p*=0.54, *I*^2^ = 0) (Figure 6(a)).

##### 3.3.2.2. In the First Week

KT significantly increased the PPT in the first week after the intervention compared with the control group (MD = 4.32, 95% CI [2.47, 6.16], *p* < 0.00001), but the data were heterogeneous (*p* < 0.00001, *I*^2^ = 97%), and this heterogeneity could not be resolved (Figure 6(b)).

##### 3.3.2.3. After 2-3 Weeks

**Figure 7:**
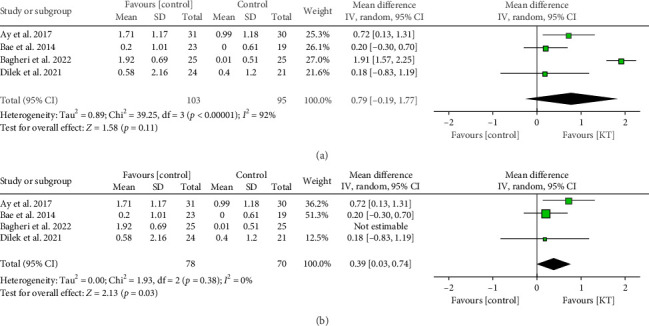
Forest plot of the change in PPT (kg/cm^2^) after 2-3 weeks. (a) Before resolving heterogeneity. (b) After resolving heterogeneity.

There was an insignificant difference between KT and control groups in the PPT after 2-3 weeks (MD = 0.79, 95% CI [−0.19, 1.77], *p*=0.11), but the data were heterogeneous (*p* < 0.00001, *I*^2^ = 92%) (Figure 7(a)). After resolving heterogeneity by excluding Bagheri et al. [30] (*p*=0.38, *I*^2^ = 0%), the data showed a significant increase in the PPT in the KT group (MD = 0.39, 95% CI [0.03, 0.74], *p*=0.03) (Figure 7(b)).

##### 3.3.2.4. After 4–6 Weeks

**Figure 8:**
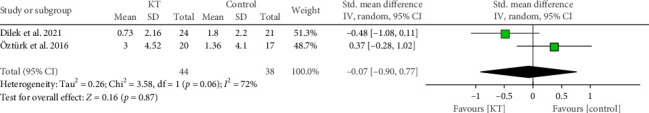
Forest plot of the change in PPT (kg/cm^2^) after 4–6 weeks.

There was an insignificant difference between KT and control groups in the PPT after 4–6 weeks (SMD = −0.07, 95% CI [−0.90, 0.77], *p*=0.87), and the data showed unresolvable heterogeneity (*p*=0.06, *I*^2^ = 72%) (Figure 8).

## 4. Discussion

The meta-analysis revealed that KT significantly reduced pain intensity in individuals with MPS immediately after the intervention, within the first week, and after 2-3 weeks when compared to the control groups. However, the effect on pain reduction diminished after 4–6 weeks, suggesting a potential need for reapplication or combined interventions for sustained long-term benefits.

The immediate and short-term pain relief observed with KT could be attributed to its proposed mechanisms of action. The elastic properties of the tape may facilitate muscle relaxation and improved blood and lymphatic circulation, reducing inflammation and muscle tension [32]. Additionally, the skin stimulation provided by the tape may modulate pain perception through the gate control theory and activate descending pain inhibitory pathways [33].

Regarding PPT, our results showed a significant increase in PPT in the KT group compared to the control group within the first week, indicating improved pain tolerance and modulation of pain processing pathways. However, this effect was not sustained beyond the first week, suggesting that the impact of KT on PPT may be short-lived.

The inconsistent findings across different time points could be attributed to several factors. The duration of tape application, the technique employed, the muscle group targeted, and individual variations in pain perception and response to treatment may all contribute to the observed heterogeneity in the results. Moreover, the potential placebo effect associated with taping should not be overlooked, as some studies utilized sham taping as a control condition.

Our findings are consistent with some previous meta-analyses that have investigated the efficacy of KT in various musculoskeletal conditions. Both our meta-analysis and the study conducted by Zhang et al. in 2019 have identified KT as effective in mitigating pain intensity compared to control groups or alternative treatments, particularly in the short term [12]. However, there are notable differences between the findings of these two studies. Our analysis revealed a significant reduction in pain scores immediately after the intervention, within the first week, and at the 2-3 weeks. However, the efficacy of KT appeared to diminish after 4–6 weeks. In contrast, Zhang et al. reported a consistent superiority of KT over other interventions at both postintervention and follow-up time points. This study encompasses comparisons with active treatments or additional therapy alongside KT, which could potentially influence the outcomes observed.

In contrast, Parreira et al. did not find evidence supporting the efficacy of this intervention in these particular clinical populations [34]. Similarly, Montalvo et al. found that KT was not superior to minimal intervention in reducing pain and disability in individuals with musculoskeletal injuries [35].

The discrepancies in findings across meta-analyses could be attributed to several factors, including differences in the populations studied, the specific conditions targeted, the control interventions used, and the methodological quality of the included studies. Additionally, variations in taping techniques, application duration, and outcome assessment methods may contribute to the inconsistencies observed.

Our meta-analysis provides important insights into the potential role of KT in the management of MPS. The significant reduction in pain intensity observed immediately after the intervention and during the first few weeks suggests that KT could be an effective adjunct therapy for short-term pain relief in individuals with MPS. Additionally, a recent RCT evaluating the epidermis dermis fascia (EDF) KT technique demonstrated significant benefits in patients with MPS affecting the upper trapezius muscle. The study found that EDF KT significantly reduced pain intensity and the number of active trigger points, while also improving cervical ROM and disability scores compared to sham KT [36]. These findings align with our meta-analytic results, reinforcing the effectiveness of KT for short-term symptom relief in MPS.

The transient effect on pain reduction observed after 4–6 weeks highlights the need for further research to investigate the optimal duration and frequency of KT application. It may be necessary to explore the combined use of KT with other interventions, such as exercise, manual therapy, or pharmacological interventions, to achieve sustained long-term benefits.

The observed increase in PPT within the first week suggests that KT may have a positive impact on modulating pain processing pathways and enhancing pain tolerance in individuals with MPS [6]. However, the lack of sustained effects on PPT beyond the first week raises questions about the underlying mechanisms and the potential need for repeated or prolonged application of KT.

The findings of this meta-analysis have important clinical implications for healthcare professionals involved in the management of MPS. KT could be considered a safe and noninvasive adjunct therapy for short-term pain relief, particularly in cases where other interventions are contraindicated or have limited effectiveness. However, it is essential to manage patient expectations and communicate the potential limitations of KT, as the effects may be temporary and require periodic reapplication or combination with other therapies [37]. Furthermore, the heterogeneity observed in the results highlights the need for additional research to explore the factors that influence the effectiveness of KT, such as taping techniques, application duration, muscle group targeted, and individual patient characteristics. Standardized protocols and rigorous methodological approaches are essential to enhance the quality and consistency of future research in this area.

One of the key strengths of our study lies in the comprehensive search strategy employed across multiple databases, ensuring a comprehensive identification of relevant studies. Additionally, the adherence to established guidelines, such as the Cochrane Handbook for Systematic Reviews and Meta-Analyses and the PRISMA guidelines, enhances the methodological rigor and transparency of our meta-analysis. Furthermore, the inclusion of only RCTs and the assessment of study quality using the Revised Cochrane Risk of Bias tool contribute to the robustness of our findings and minimize the potential for bias. Additionally, we presented results for various endpoints ranging from immediately after the intervention to 6 weeks postintervention. However, our study is not without limitations. The heterogeneity observed in some of the analyses, despite efforts to resolve it through sensitivity analyses, highlights the variability in study designs, interventions, and outcome measures among the included studies. This heterogeneity may limit the generalizability of our findings and necessitates cautious interpretation. Further research incorporating a broader range of studies and populations would enhance the generalizability of the results.

## 5. Conclusions

Our meta-analysis provides preliminary evidence that KT is effective in reducing pain intensity and increasing PPT in individuals with MPS, particularly in the immediate and short-term periods. However, the effects on pain reduction and PPT diminish over time, suggesting a need for reapplication or combination with other interventions for sustained long-term benefits. Based on these findings, we recommend considering KT as an adjunct therapy for short-term pain relief and modulation of pain processing pathways in individuals with MPS. The significant methodological limitations, including small sample sizes, high risk of bias, and lack of blinding across studies, severely limit the reliability and generalizability of these findings. However, healthcare professionals should manage patient expectations and communicate the potential limitations of KT. Future research should focus on optimizing KT application techniques, exploring the combined use of KT with other interventions, and investigating the underlying mechanisms contributing to the observed effects. Standardized protocols and rigorous methodological approaches are essential to enhance the quality and consistency of future research in this area.

## Figures and Tables

**Table 1:** Summary of the included studies.

Study ID	Site of MPS	KT	Control	Pain scale	Follow up	Conclusion
Ay et al. [22]	Cervical	I-shape, five times by intervals of 3 days for 15 days	Sham taping	VAS	2 weeks	KT resulted in improvements in pain, pressure pain threshold, and cervical range of motion, but it did not significantly impact disability in the short term
Bae [13]	Sternocleidomastoid muscle	Y-shape, three times per week for 2 weeks	No treatment	VAS	2 weeks	KT was considered a treatment technique that could be used for latent myofascial trigger points
Bagheri et al. [30]	Upper trapezius muscle	I-shape, 2 sessions with a 3-day interval	No treatment	—	10 days	In participants with active trigger points in the upper trapezius muscle, KT elevated the pressure pain threshold and decreased muscle thickness
Capó-Juan et al. [23]	Sternocleidomastoid muscle	KT, one session	Placebo	NPS	1 week	KT led to enhanced levels in cervical movements as measured by goniometry and contributed to an improvement in quality of life
Dilek et al. [29]	Upper trapezius muscle	I-shape, two times per week for 3 weeks	KT by untrained	VAS	6 weeks	KT, whether administered by trained or untrained physiatrists, resulted in improvements in pain, palpable muscle spasm, neck function, and overall quality of life in patients with MPS
El-Azeim et al. [24]	Upper trapezius muscle	I-shape, twice per week for 4 weeks	No treatment	VAS	4 weeks	KT proved to be an effective method in managing individuals with active trigger points in the upper trapezius myofascial region
Ghuiba et al. [25]	Lumbar	KT, one session	Sham taping	VAS	4 days	The combination of PRT and KT demonstrated greater effectiveness in reducing pain levels, enhancing pressure pain threshold, and improving functional disability compared to using PRT or KT alone in the treatment of patients with lumbar myofascial pain syndrome
Halski et al. [19]	Upper trapezius muscle	Star-shape, one session	Sham taping	VAS	1 day	The two groups did not affect the resting bioelectrical activity of the upper trapezius muscle and may not result in a decrease in muscle tone in the case of myofascial trigger points (MTrPs)
Hashemirad et al. [20]	Piriformis muscle	Y-shape, one session	No treatment	VAS	3 days	Application of KT may prove effective in relieving pain and increasing range of motion (ROM) in patients experiencing myofascial trigger points in the piriformis muscle
Kalichman et al. [26]	Upper trapezius and gastrocnemius muscles	I-shape, one session	Sham taping	—	1 day	Placing KT directly above the myofascial trigger points (MTrPs) may prevent an immediate increase in sensitivity (indicated by a decrease in pressure pain threshold) after application
Kardes et al. [31]	Upper trapezius muscle	Star-shape, one session	Sham taping	VAS	Immediately after intervention	Star was found to be more effective in reducing pain and increasing cervical flexion range of motion (ROM)
Noguera-Iturbe et al. [28]	Upper trapezius muscle	Star-shape, one session	Sham taping	—	3 days	The results of this study did not support the use of the Kinesio taping (KT) technique for treating patients with latent or active myofascial trigger points in the upper trapezius muscle
Öztürk et al. [21]	Upper trapezius muscle	I-shape, applied twice, with one day of rest	Sham taping	VAS	1 month	Patients with myofascial pain syndrome who received Kinesio taping exhibited statistically significant improvements in pain and upper trapezius muscle strength
Rasti and Shamsoddini [14]	Upper trapezius muscle	X-shape, applied every 3 days, for 2 weeks	Sham taping	NRS	2 weeks	KT can reduce neck pain, increase neck range of motion, and ultimately decrease the disability caused by myofascial pain syndrome both in the short term and long term
Shakeri et al. [27]	Lateral epicondylitis	Diamond shape, 3 times in 1 week	Sham taping	VAS	1 week	KT led to improvements in pain intensity and upper extremity disability in subjects with lateral epicondylitis (LE) and myofascial trigger points (MTP) in forearm muscles, with KT applied with tension being more effective than a placebo

*Note:* MTrPs = myofascial trigger points.

Abbreviations: KT = Kinesio taping, MPS = myofascial pain syndrome, NPS = numerical pain scale, NRS = numerical rating scale, PRT = positional release therapy, ROM = range of motion, VAS = visual analog scale.

**Table 2:** Baseline characteristics of included studies.

Study ID	Study arms	Sample	Age, years, mean ± SD	Sex, male, *n* (%)	BMI, kg/m^2^, mean ± SD	Pain score, mean ± SD	PPT, kg/cm^2^, mean ± SD
Ay et al. [22]	KT	31	44.80 ± 17.19	9 (22.6%)	—	VAS: 5.0 ± 2.00	6.24 ± 0.9
	Control	30	44.10 ± 17.45	7 (23.3%)	—	VAS: 4.56 ± 2.17	6.29 ± 0.55

Bae [13]	KT	23	23.3 ± 2.7	9 (39.1%)	—	VAS: 1.42 ± 0.92	5.10 ± 2.08
	Control	19	22.8 ± 3.2	8 (42.1%)	—	VAS: 1.39 ± 0.15	4.5 ± 2.4

Bagheri et al. [30]	KT	25	30.4 ± 5.68	0	23.5 ± 3.22	—	12.6 ± 3.4
	Control	25	29.7 ± 5.92	0	24.0 ± 3.50	—	13.9 ± 3.6

Capó-Juan et al. [23]	KT	25	39.12 ± 1.63	—	24.45 ± 0.93	NPS: 5.32 ± 0.42	2 ± 0.29
	Control	25	38.80 ± 1.91	—	24.25 ± 0.64	NPS: 5.36 ± 0.37	2.8 ± 0.78

Dilek et al. [29]	KT	24	32.5 ± 8.0	—	—	VAS: 4.5 ± 2	4.4 ± 1.45
	Control	21	31.3 ± 8.1	—	—	VAS: 4.5 ± 2	3.9 ± 0.9

El-Azeim et al. [24]	KT	20	23.20 ± 1.67	—	—	VAS: 7.35 ± 0.67	—
	Control	20	23.5 ± 1.76	—	—	VAS: 7.1 ± 0.72	—

Ghuiba et al. [25]	KT	15	31 ± 2.53	8 (53.3%)	28.96 ± 0.95	VAS: 8.21 ± 0.23	0.73 ± 0.18
	Control	15	31.2 ± 2.11	6 (40%)	28.58 ± 1.35	VAS: 8.21 ± 0.23	0.79 ± 0.2

Halski et al. [19]	KT	25	20.6 ± 1.5	4 (16%)	22.9 ± 3.1	VAS: 6.8 ± 1.8	—
	Control	24	19.9 ± 0.8	0	21.9 ± 3.2	VAS: 6.4 ± 1.6	—

Hashemirad et al. [20]	KT	33	42.2 ± 15.8	—	—	VAS: 6.5 ± 2.8	—
	Control	18	42.7 ± 12.7	—	—	VAS: 5.33 ± 2.7	—

Kalichman et al. [26]	KT-upper trapezius	16	25.44 ± 1.63	6 (37.5%)	21.88 ± 1.99	—	33.4 ± 14.98
	Control-upper trapezius	16	26.06 ± 1.88	5 (31.5%)	22.61 ± 2.16	—	30.27 ± 11.99
	KT-gastrocnemius	15	24.93 ± 2.02	5 (33.3%)	21.58 ± 2.49	—	23.27 ± 14.2
	Control-gastrocnemius	15	26.27 ± 1.79	5 (33.3%)	22.64 ± 3.04	—	26.9 ± 9.35

Kardes et al. [31]	KT	14	22.79 ± 2.94	2 (14.3%)	20.30 ± 6.14	VAS: 5.42 ± 1.45	4.94 ± 2.4
	Control	14	21.57 ± 2.02	1 (7.1%)	22.99 ± 3.33	VAS: 4.57 ± 1.50	4.65 ± 1.7

Noguera-Iturbe et al. [28]	KT	20	22.9 ± 7.1	6 (30%)	22.6 ± 4.5	—	2.61 ± 1
	Control	17	21.7 ± 6.2	5 (29.4%)	22.8 ± 4.1	—	2.94 ± 0.95

Öztürk et al. [21]	KT	20	29.95 ± 4.90	3 (17.7%)	22.71 ± 3.02	VAS: 6.86 ± 1.87	3.85 ± 2.62
	Control	17	33.86 ± 8.47	3 (17.7%)	22.4 ± 4.60	VAS: 6.45 ± 1.19	4.93 ± 2.53

Rasti and Shamsoddini [14]	KT	15	30.20 ± 3.55	6 (40%)	—	NRS: 4.93 ± 1.53	—
	Control	15	32.80 ± 6.98	9 (60%)	—	NRS: 5.33 ± 1.83	—

Shakeri et al. [27]	KT	15	37.6 ± 11.56	0	21.47 ± 2.31	VAS: 6.4 ± 1.99	1.62 ± 0.5
	Control	15	31.62 ± 11.43	0	22.04 ± 3.75	VAS: 6.0 ± 2.23	1.62 ± 0.5

*Note:* kg/cm^2^ = kilograms per square centimeter, M = male.

Abbreviations: BMI = body mass index, KT = Kinesio taping, MPS = myofascial pain syndrome, NPS = numerical pain scale, NRS = numerical rating scale, PPT = pressure pain threshold, VAS = visual analog scale.

## Data Availability

The data that support the findings of this study are available from the corresponding author upon reasonable request.

## Supporting Information

PRISMA 2020 Checklist.
